# HDNA methylation data-based molecular subtype classification related to the prognosis of patients with hepatocellular carcinoma

**DOI:** 10.1186/s12920-020-00770-5

**Published:** 2020-08-24

**Authors:** Hui He, Di Chen, Shimeng Cui, Gang Wu, Hailong Piao, Xun Wang, Peng Ye, Shi Jin

**Affiliations:** 1 Department of Laparoscopic Surgery, the First Affiliated Hospital of Dalian Medical University, Lianhe Road 193#, Shahekou District, Dalian, 116000 Liaoning Province China; 2 CAS Key Laboratory of Separation Science for Analytical Chemistry, Dalian Institute of Chemical Physics, Chinese Academy of Sciences, 457 Zhongshan Rd, Dalian, 116023 China; 3 Department of Hepatobiliary Surgery, the First Affiliated Hospital of Dalian Medical University, Dalian, 116000 Liaoning Province China; 4 Department of Hepatobiliary Surgery, the First Affiliated Hospital of China Medical University, Shenyang, 110042 Liaoning Province China; 5 Department of Urological Surgery, Liaoning Cancer Hospital & Institute, Shenyang, 110042 Liaoning Province China

**Keywords:** DNA methylation, Molecular subtype classification, TCGA database, Hepatocellular carcinoma

## Abstract

**Background:**

DNA methylation is a common chemical modification of DNA in the carcinogenesis of hepatocellular carcinoma (HCC).

**Methods:**

In this bioinformatics analysis, 348 liver cancer samples were collected from the Cancer Genome Atlas (TCGA) database to analyse specific DNA methylation sites that affect the prognosis of HCC patients.

**Results:**

10,699 CpG sites (CpGs) that were significantly related to the prognosis of patients were clustered into 7 subgroups, and the samples of each subgroup were significantly different in various clinical pathological data. In addition, by calculating the level of methylation sites in each subgroup, 119 methylation sites (corresponding to 105 genes) were selected as specific methylation sites within the subgroups. Moreover, genes in the corresponding promoter regions in which the above specific methylation sites were located were subjected to signalling pathway enrichment analysis, and it was discovered that these genes were enriched in the biological pathways that were reported to be closely correlated with HCC. Additionally, the transcription factor enrichment analysis revealed that these genes were mainly enriched in the transcription factor KROX. A naive Bayesian classification model was used to construct a prognostic model for HCC, and the training and test data sets were used for independent verification and testing.

**Conclusion:**

This classification method can well reflect the heterogeneity of HCC samples and help to develop personalized treatment and accurately predict the prognosis of patients.

## Background

The liver is the greatest digestive and endocrine organ in the human body, and primary HCC, which is characterized by easy metastasis and strong invasion, is one of the most commonly seen malignant tumours in the clinic, with a low 5-year survival rate [1]. HCC has become the second major malignant tumour that threatens human life [2]. In China, the leading aetiology of HCC is viral hepatitis, among which hepatitis B and hepatitis C are the most common, while alcoholic liver disease (ALD) is dominant in western countries [3]. In recent years, an increasing number of HCC cases with unknown reasons has been diagnosed, showing a younger trend [4]. In addition, with the advancement of HCC research, it has been discovered that epigenetic changes [5], including DNA methylation, histone modification and aberrant miRNA expression, play core roles in HCC genesis, wherein HCC genesis is related to abnormal DNA methylation [6]. A large amount of literature has indicated that DNA methylation represents a series of early and most frequent molecular behaviours during the HCC genesis process [7]. DNA methylation accumulates with disease progression and is currently regarded as an indication of malignant HCC [8]. Therefore, obtaining samples from HCC patients after diagnosis, carrying out specific analyses, and mining the specific biomarkers are the key to prognostic assessment, classification determination, recurrence judgement, and early selection of appropriate therapeutics and treatments.

DNA methylation refers to the process in which methyl groups are transferred onto the 5′-position carbon atom in the cytosine of the DNA CpG sequence to form 5-methylcytosine, with S-adenosylmethionine as the donor under the catalysis of DNA methyltransferases (DNMTs include three types, namely, DNMT1, DNMT2 and DNMT3) [9]. Hypermethylation of CpG islands is related to the silencing of tumour suppressor gene expression, which plays a key role during the early tumourigenesis process [10]. Additionally, the universally low methylation level of the tumour genome is closely associated with the activation of oncogenes, changes in chromatin structure, and the loss of basic groups; moreover, it is also closely correlated with tumour invasion, metastasis and prognosis [11]. Currently, research has verified that genome-wide low DNA methylation is an important mechanism responsible for early HCC formation and is also an important cause of early chromatin structural changes in non-cirrhosis HCC. The low methylation of genes such as AKT3, CD147, LINE1 and SFRP1 has been proven to be significantly correlated with the survival, tumour volume and malignant grade of HCC patients [12–14]. Moreover, the silencing of tumour suppressor genes and the loss of DNA repair function in HCC are closely correlated with the high methylation of promoter CpG islands, which prevents the timely repair of DNA damage, thus resulting in abnormal cell proliferation; for instance, the CpG islands of genes such as SOX1, VIM, BMP4 and CDKN2A show high methylation levels [15–17]. However, it is still unknown whether specific methylation patterns in these gene promoter regions display clinical significance compared with tumour classification, survival and prognosis in large-sample HCC patient data.

Therefore, this study aimed to establish a classification method that integrates several DNA methylation markers in order to help clinicians effectively assess HCC patient prognosis and select therapeutic strategies.

## Methods

### Pre-processing of preliminary sample data and initial screening of HCC DNA methylation sites

The latest clinical follow-up information was downloaded using the TCGA GDC API on July 31st, 2018 (as shown in Table S1.txt) and contained a total of 377 samples (as shown in Table S11.txt); the RNA-Seq data (as shown in Table S2.txt) included 424 samples. Illumina Infinium HumanMethylation450 data [18] were downloaded using the UCSC Cancer Browser [19] and contained 429 samples.

Subsequently, the samples with a follow-up period of over 30 days were screened from the clinical data for further methylation profile matching, and a total of 348 samples detected for methylation were screened. Moreover, the CpG sites with an NA ratio of over 70% were removed from all samples, and the cross-reactive CpG sites in the genome were also removed according to the cross-reactive sites. In addition, the missing values of methylation profiles were filled using the KNN method with the impute R package, and the unstable genomic methylation sites were further removed in order to remove the CpG sites and single nucleotide sites from the sex chromosomes. A total of 208,022 methylation sites were finally obtained. Second, the 348 samples were divided into a training set (*n* = 174) and test set (*n* = 174). The obtained clinical follow-up information is shown in Table S3.txt and the clinical follow-up information is shown in Table S4.txt.

### Univariate analysis and multivariate survival analysis of the training set methylation sites

First, a univariate Cox proportional hazards regression model was applied to analyse each methylation site and the survival data using the coxph function of the survival R package [20], with *p* < 0.05 as the significant threshold. Subsequently, the univariate Cox proportional hazards regression model was also employed to analyse age, T, N, and M stages, grade, sex and survival data. The results suggested that the T, N and M stages showed significant differences in prognosis prediction, with log-rank *p*-values of 0.002446, 0.034366 and 0.00259, respectively. Then, the univariate Cox model was used to select the significant methylation sites for multivariate Cox proportional hazards regression model analysis, with the T, N and M stages as the covariants in the model and *P* < 0.05 as the significant threshold. Finally, the methylation sites of the training set samples that were significant in both the univariate and multivariate analyses were selected as characteristic biomarkers for further analysis.

### Molecular subtyping of HCC, screening of intra-group-specific methylation sites, and enrichment analysis of signalling pathways and transcription factors

First, the ConsensusClusterPlus R software package [21] was used for the consistent clustering of characteristic methylation sites that were significant in both the univariate and multivariate analyses, and the molecular subtypes were screened for subgroup classification. The similarity distance between samples was calculated by the Euclidean distance [22], K-means was used for clustering, 80% samples were sampled 100 times by adopting the resampling program, and the optimal cluster number was determined by the cumulative distribution function (CDF) [23]. On this basis, methylation expression profile cluster analysis and clinical characteristic analysis of each subgroup were also performed. Subsequently, the EpiDiff software [24] was also employed to identify the specific methylation sites. For each cluster, the average value of each methylation level of the methylation sites that were significant in both the univariate and multivariate analyses was calculated, the obtained matrix was used as the input data of EpiDiff software, the threshold was set at 2.099, and the cluster-specific methylation sites were screened for genomic annotations. Additionally, the correlation of these specific methylation sites with the gene expression in the subgroups was explored. A total of 172 corresponding samples that had been detected with RNA-Seq were identified in the training set samples, gene expression profiles were extracted from these 172 samples to plot the expression profile heat map, and the correlation and consistency of the gene DNA methylation level with gene expression was observed. Finally, the corresponding genes in the promoter regions of these specific methylation sites were subjected to signalling pathway and transcription factor enrichment analysis using cluster Profiler [25] and g:profiler [26], respectively.

### Construction and testing of the prognosis prediction model for HCC patients

The Bayesian network classifier was constructed using the above identified specifically expressed methylation sites in each subtype, and the model performance was judged through 10-fold cross-validation. Subsequently, the expression profile data of specific CpG methylation sites were extracted from both the training set and test set and were substituted into the model for calculation. In addition, the prediction results were calculated, and the prediction accuracy of the prognosis classification model as well as the identification stability of the methylation features were verified and analysed. (A study flowchart has been added, see Figure S1.tif).

## Results

### Mining characteristic DNA methylation sites based on the survival and prognosis data of HCC patients

First, a series of data downloaded from TCGA was pre-processed, including filling in the missing values, removing the CpG sites and single nucleotide sites from the sex chromosomes, removing the CpG sites with cross-reactivity from the genome, excluding samples with incomplete data, and randomly dividing the samples into a training set and test set (see Materials and methods). Afterwards, the methylation sites and survival data were subjected to univariate Cox proportional hazards regression model analysis (using the coxph function in the survival R package, see Table S5.txt), with the significance threshold set as *P* < 0.05. A total of 26,208 significantly different sites regarding prognosis were discovered, and the top 20 are shown in Table 1. Similarly, our findings revealed that the T, N and M stages were also significantly correlated with prognosis, with log-rank *P* values of 0.002446, 0.034366 and 0.00259, respectively. In addition, the univariate Cox model was used to select the significant methylation sites for multivariate Cox proportional hazards regression model analysis, with the T, N and M stages as the covariants in the model. Finally, a total of 10,699 significant methylation sites were obtained (see Table S6.txt). As a result, the methylation sites (*n* = 10,699) from the training set samples that were significant in both the univariate and multivariate analyses were selected as characteristic biomarkers for further analysis.

**Table 1:** The top 20 most significant methylation sites

CpGs	***p***.value	HR	Low 95%CI	High 95% CI
cg11835695	1.14E-09	2.35E-08	8.24E-11	6.72E-06
cg09407273	2.32E-08	2.04E-06	2.06E-08	0.000202
cg05978187	3.70E-08	7.96E+ 50	5.98E+ 32	1.06E+ 69
cg17922226	5.45E-08	0.007683	0.001328	0.044456
cg16474647	6.07E-08	0.00037	2.12E-05	0.006453
cg13754720	7.69E-08	0.000261	1.29E-05	0.005295
cg22288195	9.21E-08	0.000397	2.25E-05	0.007031
cg16068336	1.72E-07	7.73E+ 16	3.6E+ 10	1.66E+ 23
cg14520941	1.73E-07	0.000291	1.37E-05	0.006172
cg25319233	2.20E-07	0.000656	4.10E-05	0.010496
cg07285167	2.32E-07	0.012751	0.002441	0.066611
cg22398566	2.49E-07	4.8E+ 12	72,969,412	3.16E+ 17
cg24505619	2.61E-07	0.000544	3.11E-05	0.009507
cg25841634	2.93E-07	513,128.5	3370.924	78,109,408
cg08079806	3.17E-07	0.000436	2.25E-05	0.008466
cg03236992	3.25E-07	1E+ 179	6.60E+ 79	1E+ 179
cg00121045	3.36E-07	0.000139	4.57E-06	0.004206
cg25400614	3.37E-07	1.02E-08	8.72E-12	1.20E-05
cg14826331	3.39E-07	0.020849	0.004712	0.092258
cg09169615	4.24E-07	6,102,271	14,325.15	2.6E+ 09

### Using the characteristic DNA methylation sites for the consistent clustering of HCC molecular subgroups

**Fig. 1:**
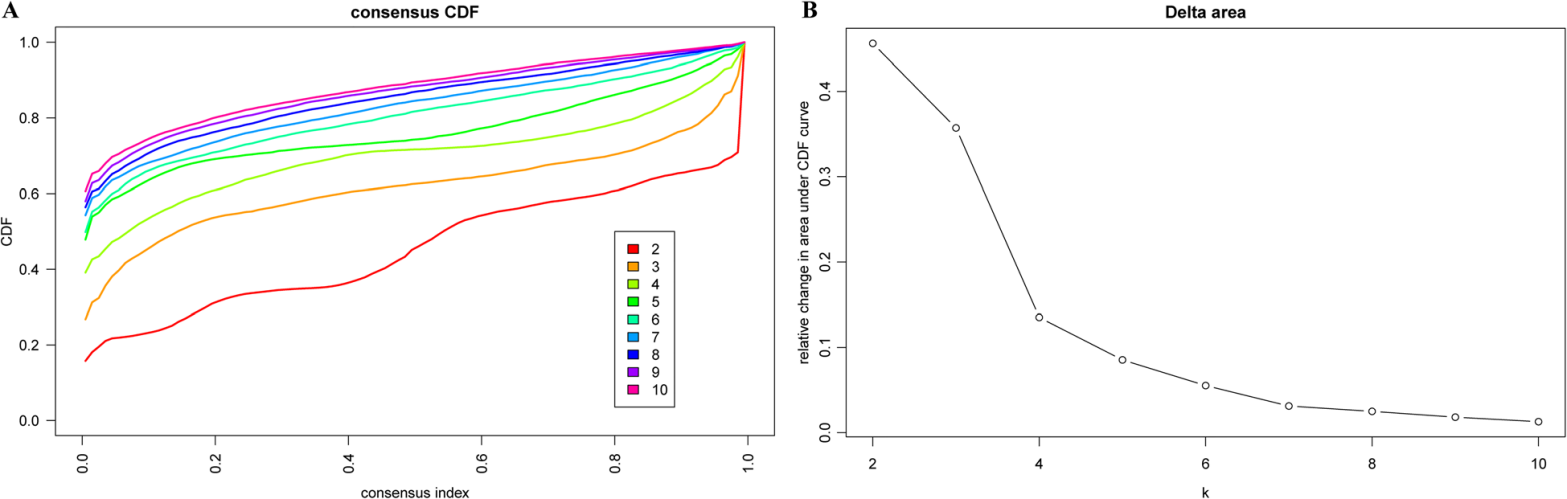
The criteria for the selection of the number of categories. **a** The consensus among clusters for each category number k. **b** Delta area curve of consensus clustering, which indicates the relative change in area under the cumulative distribution function (CDF) curve for each category number k compared to that of k-1. The horizontal axis represents the category number k, and the vertical axis represents the relative change in area under the CDF curve

The Consensus Cluster Plus R software package was used for the consistent clustering of characteristic methylation sites that were significant in both the univariate and multivariate analyses, and the molecular subtypes were screened for subgroup classification. The similarity distance between samples was calculated by the Euclidean distance, K-means was used for clustering, 80% samples were sampled 100 times by adopting the resampling program, and the optimal cluster number was determined by the CDF. As shown in Fig. 1a, stable clustering results could be obtained when the cluster number was 6 or 7. Further observation of the CDF delta area curve (Fig. 1b) suggested that stable clustering results could be acquired when the cluster number was set as 7. Finally, *k* = 7 was selected to obtain 7 molecular subtypes.

### Methylation expression profile cluster analysis and clinical characteristic analysis based on the HCC molecular subgroups

**Fig. 2:**
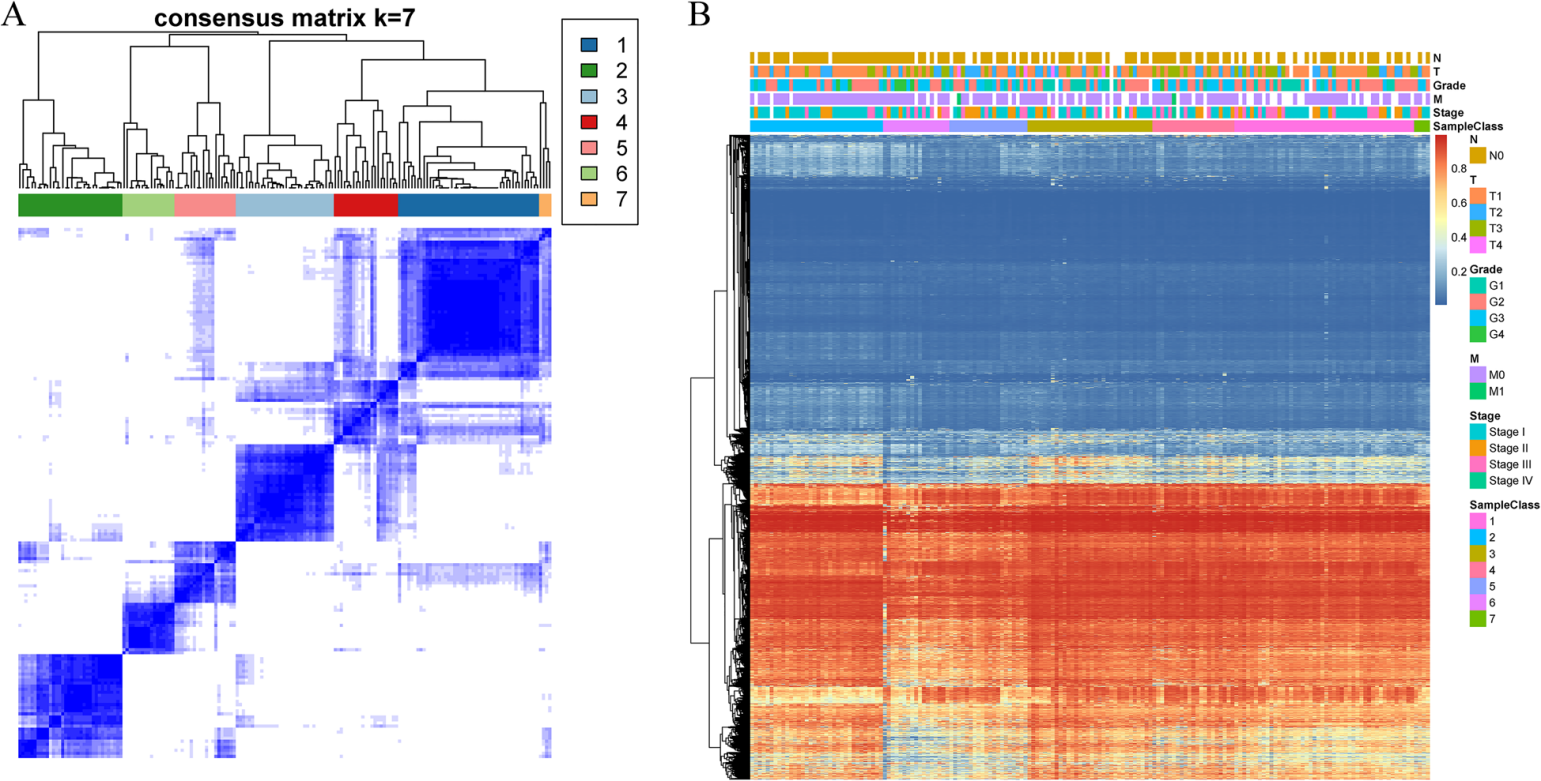
The consensus matrix for DNA methylation classification with the corresponding heat map. **a** The colour-coded heatmap corresponding to the consensus matrix for *k* = 7 obtained by applying consensus clustering. The colour gradients from 0 to 1 represent the degree of consensus, with white corresponding to 0 and dark blue corresponding to 1. **b** The heatmap corresponding to the dendrogram in Figure **a**, which was generated using the pheatmap function with DNA methylation classifications, TNM stage, clinicopathological stage and histological type as the annotations

The stable clustering results at *k* = 7 were selected according to consistent clustering. As presented in Fig. 2a, 174 tumour samples were assigned to these 7 subgroups. Furthermore, 10,699 methylation profiles were used for cluster analysis, and the distance between methylation sites was calculated by the Euclidean distance. Figure 2b shows that most methylation sites had low abundance in each sample, while the methylation expression profiles of these 7 clusters of samples were also greatly different. In particular, the methylation levels of the samples in Cluster5 and Cluster6 were markedly lower than those of the other groups.

**Fig. 3:**
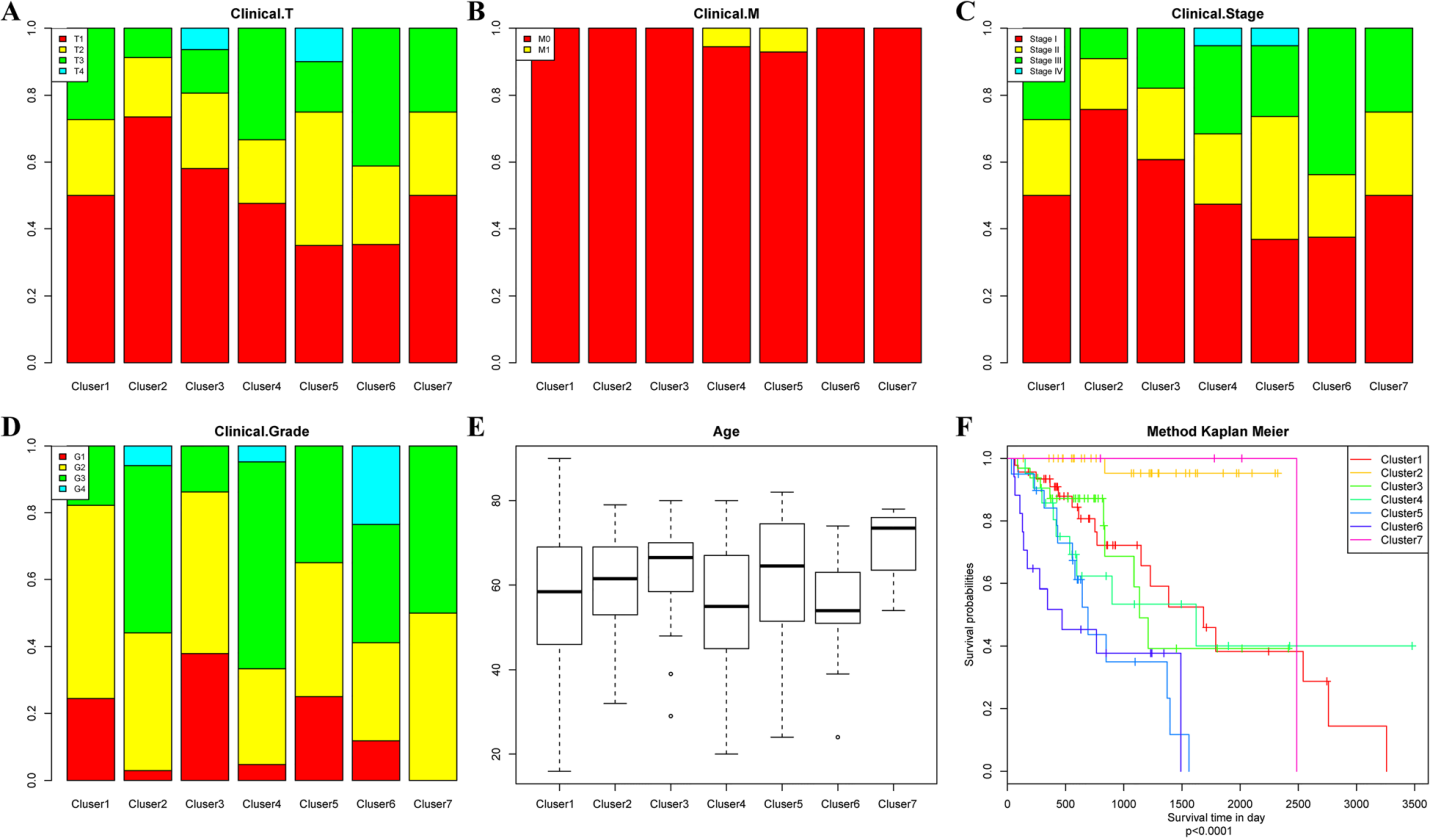
Comparison of prognosis, TNM stage, grade and age between each DNA methylation cluster. **a** The survival curves of each DNA methylation subtype in the training set. The horizontal axis represents the survival time (days), and the vertical axis represents the probability of survival. The metastasis **b**, stage score **c**, grade score **d**, age **e** and survival probability **f** distributions of each DNA methylation subtype in the training set. The horizontal axis represents the DNA methylation clusters

The distribution of the samples according to the 7 molecular subtypes regarding prognosis, T, N, and M stage, grade and age was further analysed, as displayed in Fig. 3. Figure 3a shows that the samples in Cluster5 and Cluster6 had the highest invasion degrees; Fig. 3b suggested that M1 samples were mainly distributed in Cluster4 and Cluster5; Fig. 3c indicated that the samples in Cluster5 and Cluster6 had high stages; Fig. 3d suggested that Cluster6 had the largest number of G4 samples; Fig. 3e presented the great difference in the age distribution among the different clusters; and Fig. 3f revealed a significant difference in prognosis among these 7 clusters of samples, among which the samples in Cluster2 had the best prognosis, while the samples in Cluster5 and Cluster6 had the poorest prognosis.

### Screening of intra-group-specific methylation sites and enrichment analysis of transcription factors based on the HCC molecular subtype classification

**Fig. 4:**
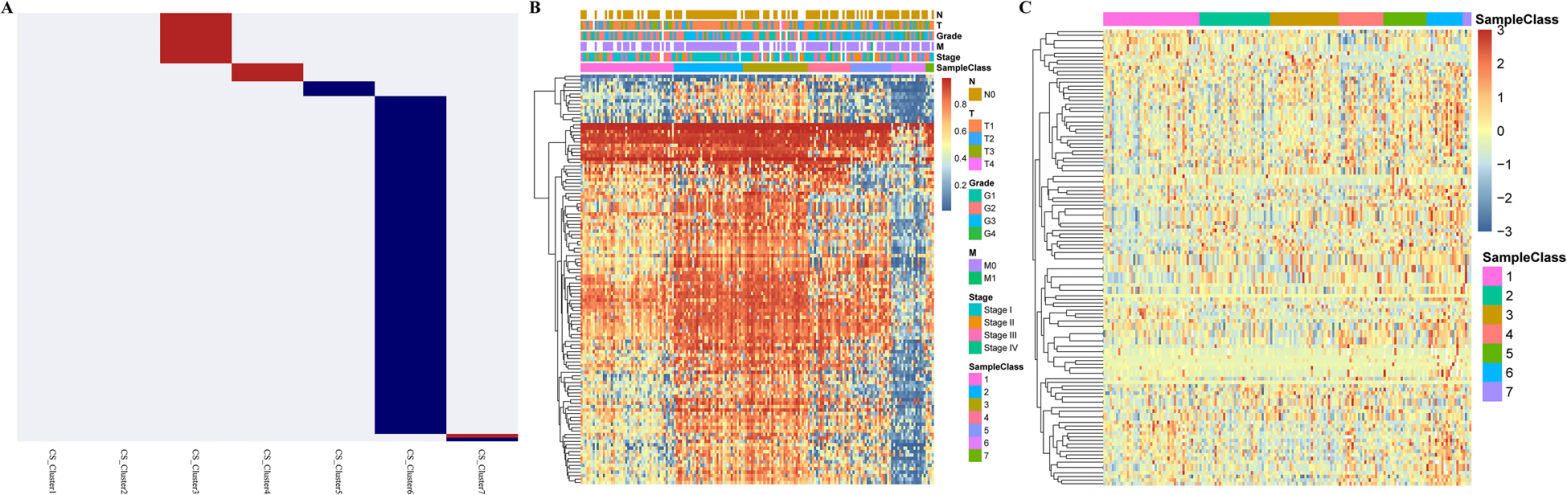
The specific hyper/hypomethylated CpG sites for each DNA methylation cluster. **a** The display of specific CpG sites for each DNA methylation prognostic subtype. The red bars and blue bars represent hypermethylated CpG sites and hypomethylated CpG sites, respectively. **b** The heat map for the specific sites among the seven DNA methylation clusters. **c** The heatmap for the expression of specific related genes among the seven DNA methylation clusters

To identify the methylation-based molecular subtypes of HCC, the EpiDiff software was used to identify specific methylation sites. For each cluster, the average value of the methylation levels of the 10,699 methylation sites was calculated, and the obtained 10,699 × 6 matrix was used as the input data of EpiDiff software, as presented in Table S7.txt. The threshold was set at 2.099, and 119 methylation sites were considered cluster-specific methylation sites, as displayed in Table S8.txt. The heat map is presented in Fig. 4a, which suggested that Cluster6 had the most specific methylation sites, and most of them were lowly methylated sites, while a small number of specific methylation sites also existed in the remaining clusters, which were mostly hypermethylated. In addition, there were no specific methylation sites in Cluster1. The modification level heat map of the specific methylation modification sites is illustrated in Fig. 4b. These 119 methylation sites were then subjected to genomic annotations, which obtained 105 genes near these methylation sites, as shown in Table S9.

Moreover, the correlation of these specific methylation sites with gene expression in the subgroups was explored. A total of 172 corresponding samples that had been detected with RNA-Seq were identified in the training set samples, 105 gene expression profiles were extracted from these 172 samples (as presented in Table S10), and the expression profile heat map was further plotted. As shown in Fig. 4c, there were different expression patterns in these subgroups at the expression profile level, suggesting partial consistency between the DNA methylation levels of these genes and their expression.

**Fig. 5:**
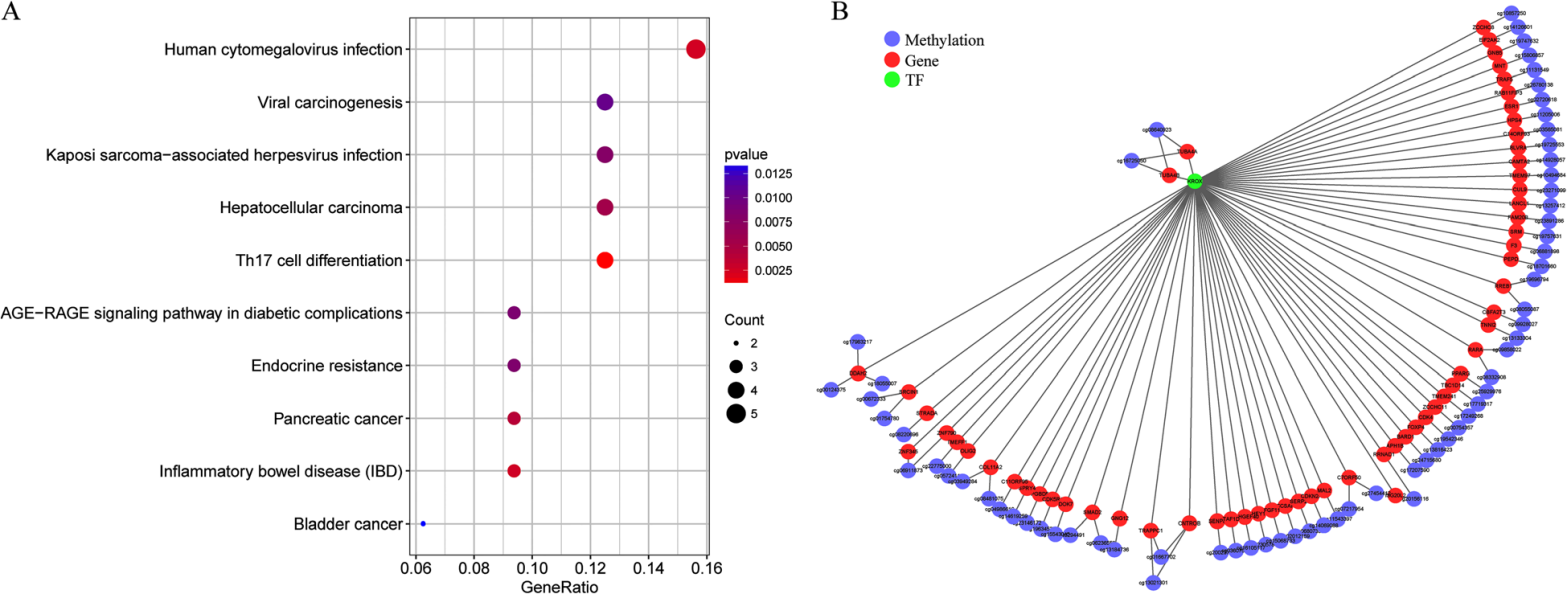
Transcription factor and KEGG pathway enrichment analyses of the specific CpG sites. **a** KEGG enrichment analysis was carried out on the corresponding genes in the promoter regions. **b** The corresponding genes were enriched by transcription factor enrichment analysis

Furthermore, to observe the mechanism of action of these specific methylation sites, KEGG enrichment analysis was carried out on the corresponding genes in the promoter regions of these specific methylation sites using the cluster Profiler R software package, as shown in Fig. 5a. It could be seen from the figure that these genes were enriched in multiple cancer-related pathways, especially the HCC pathway. Additionally, g: profiler was further used for transcription factor enrichment analysis, which revealed that these genes were enriched in the transcription factor KROX (TF: M00982), with a significant FDR value of 8.46e-03. The corresponding genes are shown in Fig. 5b, which suggested that 48 genes were enriched in KROX and involved 67 methylation sites.

### Construction of the prognosis prediction model for HCC patients and evaluation in the test set

**Fig. 6:**
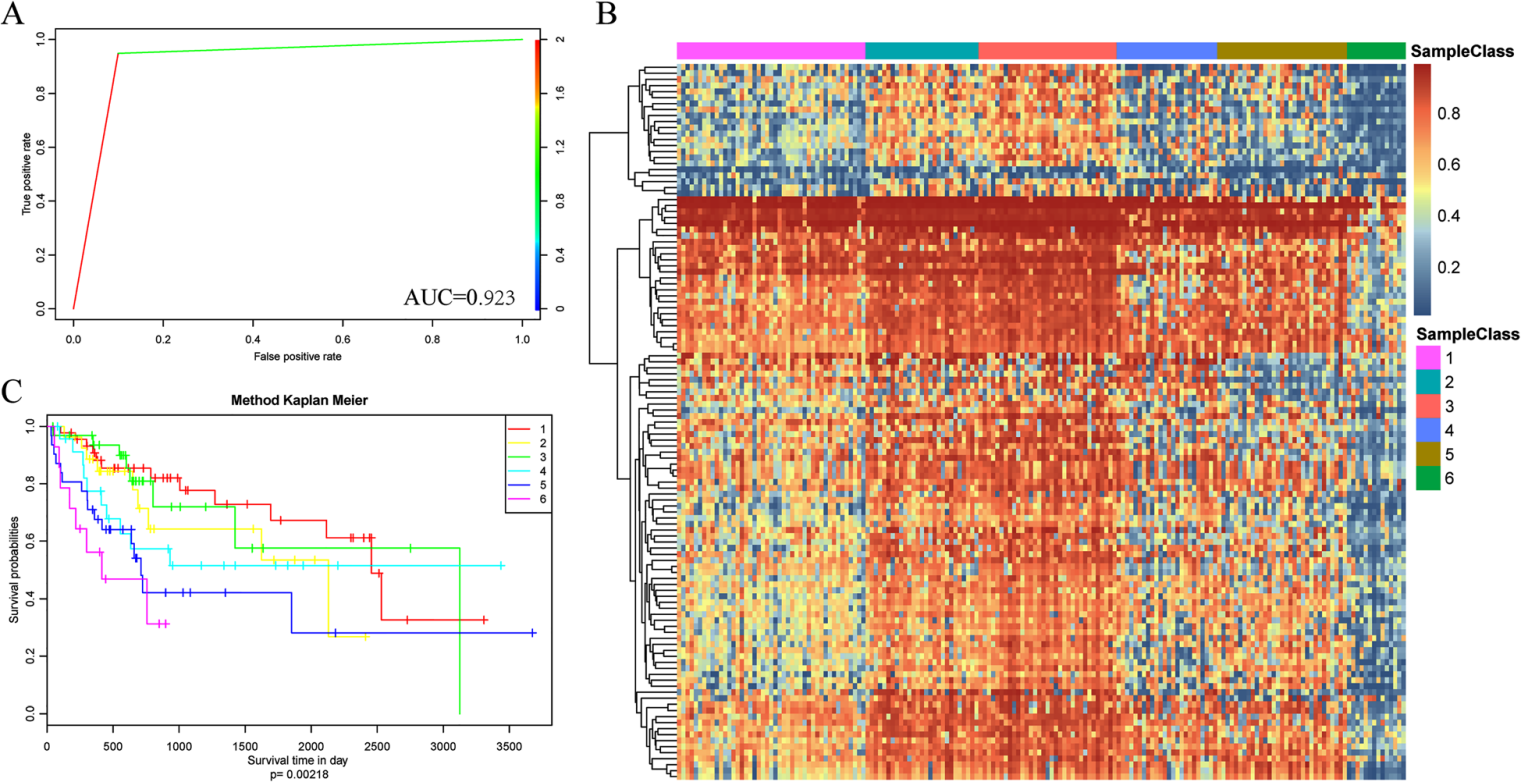
Construction of the prognosis prediction model for HCC patients. **a** The ROC curve displays the sensitivity and specificity of the prognosis prediction model. The area under the curve (AUC) reached 0.923. **b** The colour-coded heatmap corresponding to the consensus matrix for *k* = 7 obtained by applying consensus clustering from the test set. **c** Survival curves of the seven clusters predicted from the test set using the prognosis model. The log-rank test was used to assess the statistical significance of the difference

To verify the differential ability of the identified specific methylation sites, the 119 specific methylation sites identified using EpiDiff software were used to construct a Bayesian network classifier, and the model performance was judged through 10-fold cross-validation. The classification accuracy of the model constructed based on the training set was 70.68%, and the area under the ROC curve (AUC) was 0.923 (Fig. 6a), suggesting that this classifier had a favourable classification performance.

Furthermore, to verify the stability and reliability of the model, the expression profile data of these 119 CpG methylation sites were extracted from the test set and incorporated into the model for model verification. The predicted results are shown in Table 2, which shows that the cluster statistic number of the training set was similar to that of the predicted samples. Furthermore, the methylation patterns of these 7 clusters of samples were plotted using the pheatmap R package (Fig. 6b). The heatmap showed the presence of markedly distinct methylation patterns among these 7 clusters of samples. Moreover, the differences in prognosis among these 7 clusters of samples were also analysed (Fig. 6c), and significant differences were found in the prognosis of these 7 clusters of samples (*p* = 0.00068). In addition, the Cluster2 samples showed markedly better prognoses over the other clusters of samples, which were consistent with the findings in the training set data.

**Table 2:** Statistics of various samples predicted in the test set

Cluster	Number of Samples
1	45
2	27
3	33
4	24
5	31
6	14
7	0

**Fig. 7:**
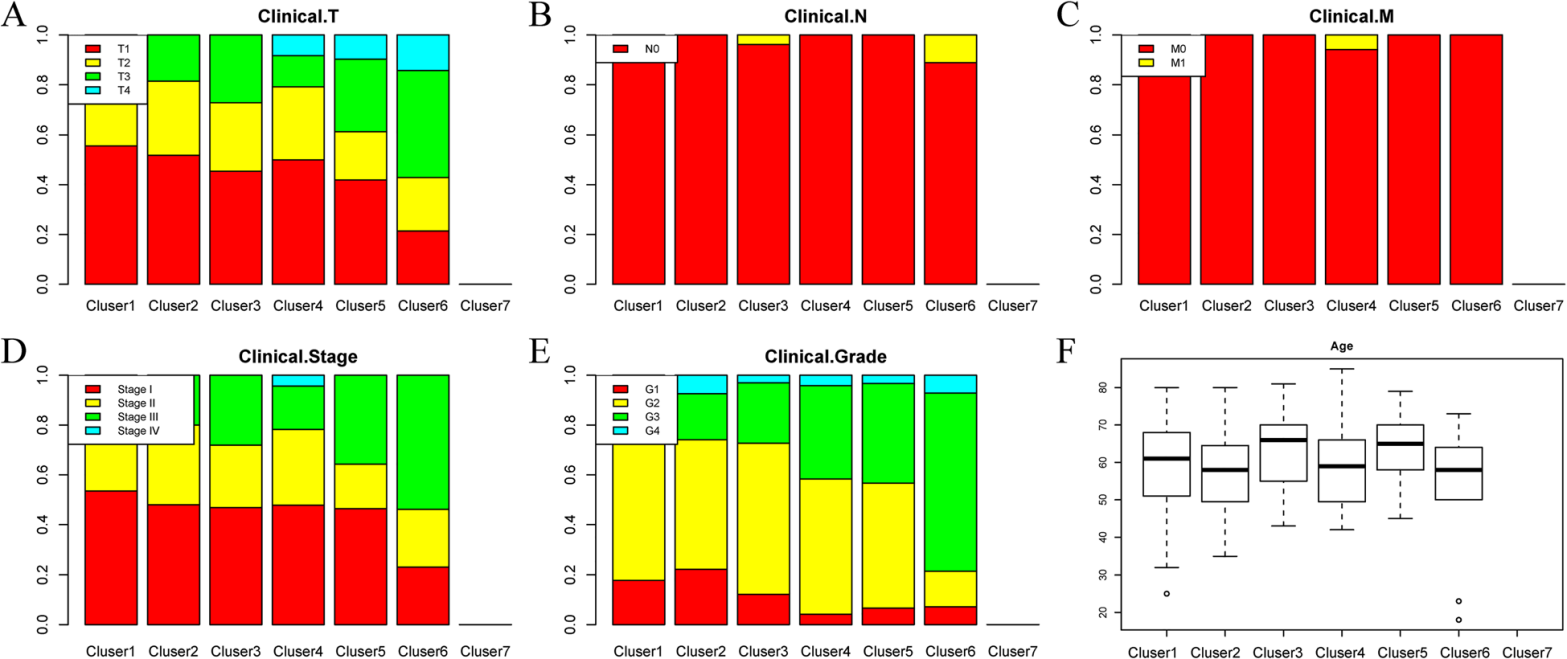
Verification of the stability and reliability of the prognosis prediction model for HCC patients in the test set. The topography score (**a**), lymphocyte infiltration (**b**), metastasis (**c**), stage score (**d**), grade score (**e**) and age (**f**) distributions of the DNA methylation clusters in the test set

Finally, the clinical features of these 7 clusters of samples in the test dataset were analysed, and Fig. 7 shows that the clinical feature distribution in each cluster was consistent with that in the training set. Overall, the prognosis prediction model constructed using these 119 methylation profiles showed high prediction accuracy and stability in identifying the methylation features.

## Discussion

HCC is one of the most commonly seen malignant tumours and is the third leading the global digestive system tumour in terms of its mortality; the morbidity and mortality of HCC also show increasing trends [27, 28]. The leading causes of HCC are chronic hepatitis viral infection (such as HBV and HCV), liver cirrhosis, malnutrition, toxin invasion and metabolic disturbance. Currently, great achievements have been attained in the diagnosis and treatment of HCC, but its pathogenesis remains incompletely elucidated.

Studies have shown that tumours are highly heterogeneous [29], and heterogeneity is the main cause of the inconsistent clinical treatment of tumours. Finn et al. showed different clinical outcomes of different molecular subtypes of HCC, and different molecular subtypes in tumours respond differently to clinical therapeutic drugs [30]. At present, epigenetic abnormalities have been considered to be characteristic of many types of cancer [31]. The epigenome map can be used to identify the molecular subtypes of a particular cancer and is related to clinical outcomes [32]. For example, the CpG island methylation phenotype is found in many types of cancer, abnormal DNA methylation patterns can be used to detect the presence of precancerous lesions or malignant cells in tissue sections [33–35]. Abnormal DNA methylation can also be used to detect free tumour DNA in the blood of some cancer patients [36]. Seven different molecular subtypes were found in our study. They not only have different epigenetic patterns but also have significant differences in clinical characteristics such as age, tumour stage, histological type, and prognosis. These results indicate that the individuals in these clusters may have different responses to clinical drugs and provide a reference for personalized therapy and drug development. We further identified subtype-specific methylation sites that can be used as markers to predict patient outcomes.

At present, a large amount of literature has reported the role of DNA methylation in HCC diagnosis, treatment and prognostication. First, compared with genetic mutations, DNA methylation changes are earlier events during the cell carcinogenesis process, while the DNA decomposed after tumour tissue necrosis will enter the peripheral blood (plasma or serum) or other body fluids (such as saliva or sputum). Therefore, detecting the methylation status of the isolated tumour-related genes in body fluids may be applied in the early diagnosis of HCC. For instance, the promoter methylation status of two factors, TFPI2 and IGFBP7, in serum is markedly higher than that in normal subjects and hepatitis patients and may thereby become a non-invasive molecular biomarker for the early diagnosis of HCC in the clinic [37, 38]. Second, DNA methylation is mediated by methyltransferase, and it has become a novel approach in the field of HCC treatment to explore therapeutic strategies based on changes in DNMT activity and DNA methylation patterns. In addition, preclinical studies have verified that drugs targeting the DNMT action substrate (azacitidine) and the DNMT cofactor SAM (sinefungin) as well as the anti-SAM metabolite neplanosin, and the non-competitive non-nucleotide transmethylase inhibitor procainamide can reverse the abnormal methylation in tumour cells, re-express the inactivated genes, and suppress cancer cell proliferation and invasion [39–41]. Finally, DNA methylation also plays an important role in the prognosis of HCC patients. The CpG island methylation phenotype (CIMP) refers to the presence of methylation in the CpG island of multiple gene promoter regions. Some articles have suggested that patients with high tumour CIMP are associated with a markedly lower survival rate than those with low or no CIMP, while patients with various CIMP degrees also have evidently different tumour metastasis and TNM stages [42]. In addition, the correlation between the methylation of multiple gene promoter sequences and patient prognosis has also been verified, such as Tip30, RASSF1A and CD147 [43].

Nevertheless, the specific methylation sequences in the promoter regions of these gene remain unclear, and it is still unknown whether the methylation of these genes displays clinical significance compared with tumour classification, survival and prognosis in large-sample HCC patient data. This study attempted to establish a classification method that integrates several DNA methylation markers based on solving these problems in order to help clinicians effectively assess the prognosis of HCC patients and select the appropriate therapeutic strategies for HCC patients.

## Conclusions

This study identified specific methylation sites based on the TCGA database and serial bioinformatics methods at the prognosis level and constructed a prognosis prediction model for HCC patients. This model contributes to clinically identifying novel HCC markers, providing multiple targets for the precision medicine of HCC, and more accurately subtyping HCC patients at the molecular subtype level. More importantly, this model is promising for providing certain assistance and guidance for clinicians regarding prognosis judgement, clinical diagnosis and medication for HCC patients with different epigenetic subtypes.

## Supplementary information

**Additional file 1: Table S1.** HCC clinical information from TCGA database. The clinical follow-up information was downloaded from the TCGA GDC API.

**Additional file 2: Table S2.** HCC RNA-Seq data from TCGA database. RNA-Seq data included 424 HCC samples.

**Additional file 3: Table S3.** Clinical follow-up information from training set. The 348 HCC samples were divided into a training set (*n* = 174) which obtained clinical follow-up information.

**Additional file 4: Table S4.** Clinical follow-up information from test set. The 348 HCC samples were divided into a test set (*n* = 174) which obtained clinical follow-up information.

**Additional file 5: Table S5.** Differentially expressed sites were analysed by univariate Cox model. A total of 26,208 methylation sites and survival data were subjected to univariate Cox proportional hazards regression model analysis.

**Additional file 6: Table S6.** Differentially expressed sites were analysed by multivariate Cox model. A total of 10,699 significant methylation sites were obtained by multivariate Cox proportional hazards regression model analysis, with the T, N and M stages as the covariants in the model.

**Additional file 7: Table S7.** Identifying the methylation-based molecular subtypes. To identify methylation-based molecular subtype sites, the obtained 10,699 × 6 matrix was used as the input data of EpiDiff software.

**Additional file 8: Table S8.** 7 cluster-specific methylation sites. One hundred nineteen methylation sites were considered cluster-specific methylation sites.

**Additional file 9: Table S9.** Genome annotation of methylation sites. One hundred nineteen methylation sites were subjected to genomic annotations which obtained 105 genes near these methylation sites.

**Additional file 10: Table S10.** Correlation of specific methylation sites with gene expression. 105 gene expression profiles were extracted from 172 samples which were identified in the training set samples.

**Additional file 11: Table S11.** The web link of each HCC sample. The web link of each sample was supplied.

**Additional file 12.**

## Data Availability

The data was downloaded from http://xena.ucsc.edu/and https://portal.gdc.cancer.gov/. The direct web links to each of the datasets are provided in Table S11.

## Abbreviations

CpGs: CpG sites
HCC: Hepatocellular carcinoma
ALD: Alcoholic liver disease
TCGA: The Cancer Genome Atlas
AUC: Area under the ROC curve
CDF: Cumulative distribution function
CIMP: CpG island methylation phenotype

## References

[1] Singal AG, Murphy CC. Hepatocellular carcinoma: a roadmap to reduce incidence and future burden. J Natl Cancer Inst. 2019;111(6):527–8. 10.1093/jnci/djy184 PMC6579741 30544135

[2] Baecker A, Liu X, La Vecchia C, Zhang ZF (2018). Worldwide incidence of hepatocellular carcinoma cases attributable to major risk factors. Eur J Cancer Prev.

[3] Mancebo A, Varela M, Gonzalez-Dieguez ML, Navascues CA, Cadahia V, Mesa-Alvarez A, Rodrigo L, Rodriguez M (2018). Incidence and risk factors associated with hepatocellular carcinoma surveillance failure. J Gastroenterol Hepatol.

[4] White DL, Thrift AP, Kanwal F, Davila J, El-Serag HB (2017). Incidence of hepatocellular carcinoma in all 50 United States, from 2000 through 2012. Gastroenterology.

[5] Nakamura M, Chiba T, Kanayama K, Kanzaki H, Saito T, Kusakabe Y, Kato N. Epigenetic dysregulation in hepatocellular carcinoma: an up-to-date review. Hepatol Res. 2019;49(1):3–13. 10.1111/hepr.13250 30238570

[6] Fan G, Tu Y, Chen C, Sun H, Wan C, Cai X (2018). DNA methylation biomarkers for hepatocellular carcinoma. Cancer Cell Int.

[7] Sun XJ, Wang MC, Zhang FH, Kong X (2018). An integrated analysis of genome-wide DNA methylation and gene expression data in hepatocellular carcinoma. FEBS Open Bio.

[8] Cheng J, Wei D, Ji Y, Chen L, Yang L, Li G, Wu L, Hou T, Xie L, Ding G (2018). Integrative analysis of DNA methylation and gene expression reveals hepatocellular carcinoma-specific diagnostic biomarkers. Genome Med.

[9] Villanueva A, Portela A, Sayols S, Battiston C, Hoshida Y, Mendez-Gonzalez J, Imbeaud S, Letouze E, Hernandez-Gea V, Cornella H (2015). DNA methylation-based prognosis and epidrivers in hepatocellular carcinoma. Hepatology.

[10] Udali S, Guarini P, Ruzzenente A, Ferrarini A, Guglielmi A, Lotto V, Tononi P, Pattini P, Moruzzi S, Campagnaro T (2015). DNA methylation and gene expression profiles show novel regulatory pathways in hepatocellular carcinoma. Clin Epigenetics.

[11] Udali S, Guarini P, Moruzzi S, Ruzzenente A, Tammen SA, Guglielmi A, Conci S, Pattini P, Olivieri O, Corrocher R (2015). Global DNA methylation and hydroxymethylation differ in hepatocellular carcinoma and cholangiocarcinoma and relate to survival rate. Hepatology.

[12] Fornari F, Milazzo M, Chieco P, Negrini M, Marasco E, Capranico G, Mantovani V, Marinello J, Sabbioni S, Callegari E (2012). In hepatocellular carcinoma miR-519d is up-regulated by p53 and DNA hypomethylation and targets CDKN1A/p21, PTEN, AKT3 and TIMP2. J Pathol.

[13] Kong LM, Liao CG, Chen L, Yang HS, Zhang SH, Zhang Z, Bian HJ, Xing JL, Chen ZN (2011). Promoter hypomethylation up-regulates CD147 expression through increasing Sp1 binding and associates with poor prognosis in human hepatocellular carcinoma. J Cell Mol Med.

[14] Huang Y, Wei L, Sun AM, Zhao RC, Zhang J, Yang HT, Li B, Sun CJ, Ding XQ, Gao B (2016). The Evaluation Value of Methylation Status of CpG Island of SFRP1 and LINE1 Gene Promoter Area in the Prognosis of Hepatocellular Carcinoma. Sichuan Da Xue Xue Bao Yi Xue Ban.

[15] Liu XY, Fan YC, Gao S, Zhao J, Chen LY, Li F, Wang K (2017). Methylation of SOX1 and VIM promoters in serum as potential biomarkers for hepatocellular carcinoma. Neoplasma.

[16] Qiu X, Hu B, Huang Y, Deng Y, Wang X, Zheng F (2016). Hypermethylation of ACP1, BMP4, and TSPYL5 in hepatocellular carcinoma and their potential clinical significance. Dig Dis Sci.

[17] Zhou Y, Wang XB, Qiu XP, Shuai Z, Wang C, Zheng F (2018). CDKN2A promoter methylation and hepatocellular carcinoma risk: a meta-analysis. Clin Res Hepatol Gastroenterol.

[18] Price ME, Cotton AM, Lam LL, Farre P, Emberly E, Brown CJ, Robinson WP, Kobor MS (2013). Additional annotation enhances potential for biologically-relevant analysis of the Illumina Infinium HumanMethylation450 BeadChip array. Epigenetics Chromatin.

[19] Goldman M, Craft B, Swatloski T, Cline M, Morozova O, Diekhans M, Haussler D, Zhu J (2015). The UCSC Cancer genomics browser: update 2015. Nucleic Acids Res.

[20] Zhang Y, Li H, Zhang W, Che Y, Bai W, Huang G (2018). LASSObased CoxPH model identifies an 11lncRNA signature for prognosis prediction in gastric cancer. Mol Med Rep.

[21] Wilkerson MD, Hayes DN (2010). ConsensusClusterPlus: a class discovery tool with confidence assessments and item tracking. Bioinformatics.

[22] van Hemert F, Jebbink M, van der Ark A, Scholer F, Berkhout B (2018). Euclidean distance analysis enables nucleotide skew analysis in viral genomes. Comput Math Methods Med.

[23] Sherman CD, Portier CJ (2000). Calculation of the cumulative distribution function of the time to a small observable tumor. Bull Math Biol.

[24] Zhang Y, Su J, Yu D, Wu Q, Yan H (2013). EpiDiff: entropy-based quantitative identification of differential epigenetic modification regions from epigenomes. Conf Proc IEEE Eng Med Biol Soc.

[25] Yu G, Wang LG, Han Y, He QY (2012). clusterProfiler: an R package for comparing biological themes among gene clusters. Omics.

[26] Reimand J, Arak T, Adler P, Kolberg L, Reisberg S, Peterson H, Vilo J (2016). g:Profiler-a web server for functional interpretation of gene lists (2016 update). Nucleic Acids Res.

[27] Endeshaw M, Hallowell BD, Razzaghi H, Senkomago V, McKenna MT, Saraiya M. Trends in liver cancer mortality in the United States: dual burden among foreign- and US-born persons. Cancer. 2019;125(5):726–34. 10.1002/cncr.31869 PMC6681907 30480828

[28] Chung W, Jo C, Chung WJ, Kim DJ (2018). Liver cirrhosis and cancer: comparison of mortality. Hepatol Int.

[29] Jeng KS, Chang CF, Jeng WJ, Sheen IS, Jeng CJ (2015). Heterogeneity of hepatocellular carcinoma contributes to cancer progression. Crit Rev Oncol Hematol.

[30] Finn RS, Aleshin A, Dering J, Yang P, Ginther C, Desai A, Zhao D, von Euw E, Busuttil RW, Slamon DJ (2013). Molecular subtype and response to dasatinib, an Src/Abl small molecule kinase inhibitor, in hepatocellular carcinoma cell lines in vitro. Hepatology.

[31] Lim S, Metzger E, Schule R, Kirfel J, Buettner R (2010). Epigenetic regulation of cancer growth by histone demethylases. Int J Cancer.

[32] Yu XD, Guo ZS (2010). Epigenetic drugs for cancer treatment and prevention: mechanisms of action. Biomol Concepts.

[33] Shigeyasu K, Nagasaka T, Mori Y, Yokomichi N, Kawai T, Fuji T, Kimura K, Umeda Y, Kagawa S, Goel A (2015). Clinical significance of MLH1 methylation and CpG Island Methylator phenotype as prognostic markers in patients with gastric Cancer. PLoS One.

[34] Karpinski P, Pesz K, Sasiadek MM (2017). Pan-cancer analysis reveals presence of pronounced DNA methylation drift in CpG island methylator phenotype clusters. Epigenomics.

[35] Kawasaki T, Ohnishi M, Suemoto Y, Kirkner GJ, Liu Z, Yamamoto H, Loda M, Fuchs CS, Ogino S (2008). WRN promoter methylation possibly connects mucinous differentiation, microsatellite instability and CpG island methylator phenotype in colorectal cancer. Mod Pathol.

[36] Galanopoulos M, Tsoukalas N, Papanikolaou IS, Tolia M, Gazouli M, Mantzaris GJ (2017). Abnormal DNA methylation as a cell-free circulating DNA biomarker for colorectal cancer detection: a review of literature. World J Gastrointest Oncol.

[37] Sun FK, Fan YC, Zhao J, Zhang F, Gao S, Zhao ZH, Sun Q, Wang K (2013). Detection of TFPI2 methylation in the serum of hepatocellular carcinoma patients. Dig Dis Sci.

[38] Akiel M, Guo C, Li X, Rajasekaran D, Mendoza RG, Robertson CL, Jariwala N, Yuan F, Subler MA, Windle J (2017). IGFBP7 deletion promotes hepatocellular carcinoma. Cancer Res.

[39] Li X, Guo H, Wang J, Wu Q, Lin X (2010). Fabrication of novel hepatoma-targeting microdisks by hydrogen bond-assisted self-assembly of an azacitidine-conjugating amphiphilic random copolymer. Acta Biomater.

[40] Jiang BG, Wang N, Huang J, Yang Y, Sun LL, Pan ZY, Zhou WP (2017). Tumor SOCS3 methylation status predicts the treatment response to TACE and prognosis in HCC patients. Oncotarget.

[41] Tran DDH, Koch A, Allister A, Saran S, Ewald F, Koch M, Nashan B, Tamura T (2016). Treatment with MAPKAP2 (MK2) inhibitor and DNA methylation inhibitor, 5-aza dC, synergistically triggers apoptosis in hepatocellular carcinoma (HCC) via tristetraprolin (TTP). Cell Signal.

[42] Wang Q, Wang G, Liu C, He X (2018). Prognostic value of CpG island methylator phenotype among hepatocellular carcinoma patients: A systematic review and meta-analysis. Int J Surg.

[43] Cheng Y, Zhang C, Zhao J, Wang C, Xu Y, Han Z, Jiang G, Guo X, Li R, Bu X (2010). Correlation of CpG island methylator phenotype with poor prognosis in hepatocellular carcinoma. Exp Mol Pathol.

