# Supplementary figures and images for: HDNA methylation data-based molecular subtype classification related to the prognosis of patients with hepatocellular carcinoma
Source: BMC Med Genomics. 2020 Aug 24;13:118. doi: 10.1186/s12920-020-00770-5 (PMC7447581; doi:10.1186/s12920-020-00770-5)

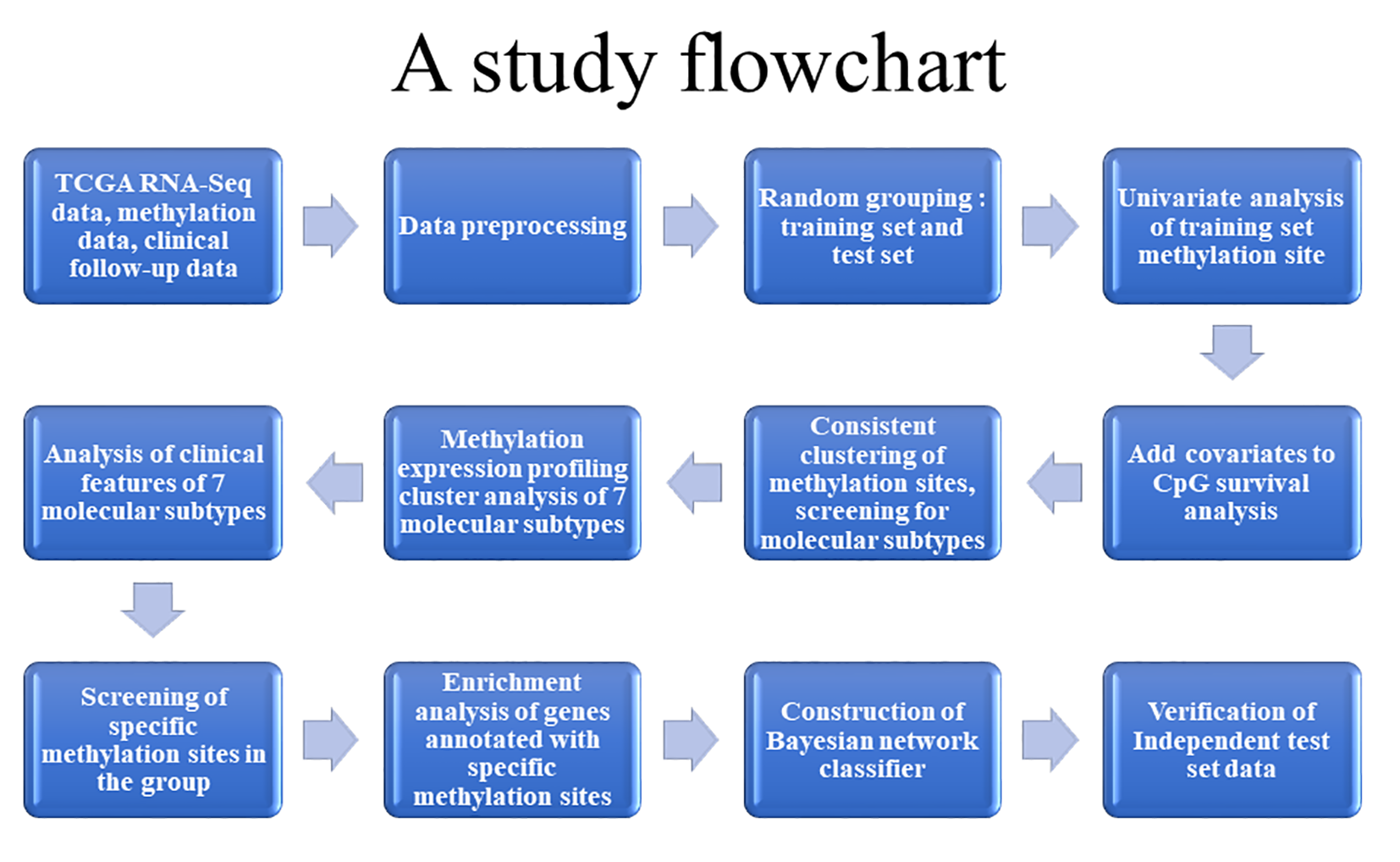

Supplement: Supplementary file 12 — Additional file 12. [file 12920_2020_770_MOESM12_ESM.tif]
